# Barriers in Rubber Dam Isolation Behaviour of Dental Students During Adhesive Restorative Treatments: A Cross-Sectional Study

**DOI:** 10.7759/cureus.58329

**Published:** 2024-04-15

**Authors:** Ahmed M. Bokhari, Thilla Sekar Vinothkumar, Nassreen Albar, Syed Nahid Basheer, Gnanasekaran Felsypremila, Waad F Khayat, Bassam Zidane, Renugalakshmi Apathsakayan

**Affiliations:** 1 Department of Preventive Dental Sciences, Division of Community Dentistry, College of Dentistry, Jazan University, Jazan, SAU; 2 Department of Restorative Dental Sciences, Division of Operative Dentistry, College of Dentistry, Jazan University, Jazan, SAU; 3 Department of Clinical Research, Sri Ramachandra Faculty of Clinical Research, Sri Ramachandra Institute of Higher Education and Research, Chennai, IND; 4 Department of Restorative Dentistry, College of Dentistry, Umm Al-Qura University, Makkah, SAU; 5 Department of Restorative Dentistry, Faculty of Dentistry, King Abdulaziz University, Jeddah, SAU; 6 Department of Preventive Dental Sciences, Division of Pedodontics, College of Dentistry, Jazan University, Jazan, SAU

**Keywords:** com-b, adhesive restoration, barriers, undergraduate students, rubber dam isolation

## Abstract

Purpose: There are unfavorable opinions connected with rubber dam isolation amongst dental students during adhesive restorative treatments. The aim of this study was to investigate the various barriers to practicing rubber dam isolation during dental procedures and provide necessary insight towards implementation of rubber dam among undergraduate dental students in Jazan.

Materials and methods: A pre-validated questionnaire in English entitled Rubber Dam Isolation Survey (E-RDIS) based on the Capability Opportunity Motivation-Behaviour (COM-B) model of behavioral change wheel was responded by 226 university dental students.

Results: The satisfaction of training was highest among sixth year students (Mean=3.57, p<0.001). Fourth year dental students scored higher in the capability (Mean=3.18) and were more highly motivated to use rubber dams (Mean=4.21). Third year students were more likely to use rubber dams in anterior teeth (Mean=3.52) whereas fourth year students use rubber dam in posterior teeth (Mean=3.74). Lack of motivation was found to be the significant barrier influencing rubber dam usage (odds ratio (OR)=12.1; 3.74, p<0.05).

Conclusion: The satisfaction with training differed among the students of different years. The rubber dam technique might be used more frequently if it were made clear to students that mastering it would be necessary for them to receive good grades.

## Introduction

Pit and fissure sealants are the preferred choice among various restorative materials due to their effectiveness in preventing further caries, efficient bonding to the tooth interface, and superior remineralization properties [1]. A rubber dam is an essential component of modern dentistry, though there were arguable questions on the endurance of the restoration favoured by the rubber dam placement [2]. A multitude of advantages was cited, including improved operator access and visibility, minimized aerosol formation, and patient safety [3,4]. It was further entrenched that rubber dam is imperative in providing isolation of the working area, standard of care, and avoiding possible risk to the patients due to fortuitous ingestion of dental instruments or restorative materials during their undergraduate years [5]. Earlier studies have shown that there are negative perceptions associated with its use among dental students [6,7]. According to a study by Mala et al., more than 50% of the interviewed dental students believed that they would be using rubber dam rarely when they start practicing independently [7]. This emphasizes the need of accentuating the use of rubber dams in clinical dentistry among future dental practitioners while they are at dental school [7].

It is important to identify various barriers behind the student’s adherence to rubber dam application and reform the guidelines to overcome those hindrances so that the students develop a positive attitude towards rubber dam isolation especially while restoring the teeth with adhesive materials. The behavioural sciences are useful in that they offer frameworks for comprehending the occurrence of behaviour, how it persists, and how to modify it [8]. The Behaviour Change Wheel served as the basis for the COM-B model, a crucial framework [9]. It is a visual depiction of a consensus study carried out by professionals in the implementation sciences and health psychology. The COM-B model describes how behaviours are caused, how interventions work, and which policy categories they fall under [10]. Thus, this model provides insight into behaviour (B) by highlighting three interconnected factors that may be changed to modify it: Capability (C), Opportunity (O), and Motivation (M) [8-10]. These three factors were considered as barriers if they promote the positive outcome behaviour and enablers if they discourage the outcome behaviour. Accordingly, Abreu-Placeres et al. [3] have introduced a validated questionnaire namely Rubber Dam Isolation Survey (E-RDIS) exclusively for adhesive restorations in operative dentistry.

Acceptable behaviour in dental students involves strict rubber dam implementation in all cases unless absolutely contraindicated. The COM-B model provides an empirically supported approach to pinpoint the elements that require modification in order to alter undesirable behaviours [3,8,9]. Identifying the barriers will help the stakeholders in modifying policies that will help students understand the importance of rubber dam usage and ultimately increase its usage in clinical dentistry. Therefore, the aim of this study was to investigate the various barriers in practicing rubber dam isolation while performing dental procedures and provide necessary insight into it among clinical undergraduate dental students.

## Materials and methods

The study was conducted in accordance with the Declaration of Helsinki, and the protocol was approved (Ref. No.: CODJU 2103F) by the Institutional Review Board of Jazan University.

Sample size and participants

The sample size analysis was utilized using G*Power statistical program software (Version 3.1.9.3; Heinrich-Heine-University, Düsseldorf, Germany) with alpha value = 0.05, beta = 0.80, two-tailed, the effect size was medium (0.03), and the suggested sample size was 200 participants. Dental students of both genders in their third, final, fifth, sixth year and interns who attended operative dentistry classes were eligible for participating in this study. First and second year students who did not take any operative dentistry classes were excluded from the study. The rest of class year students and interns were included in the study.

Questionnaire

**Figure 1:**
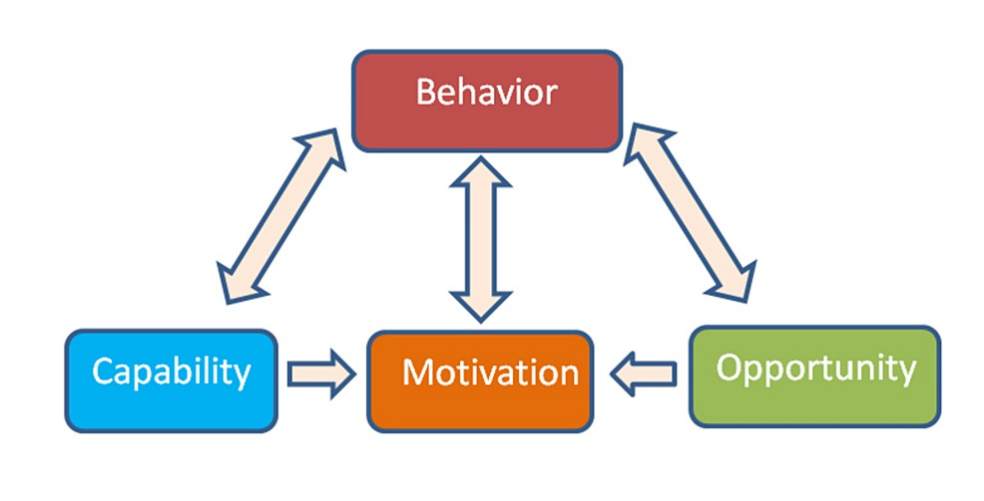
Interaction between the components of the path COM-B model associated with the RDIS items The image has been recreated based on the idea from the article by Michie et al. [9]. The single-headed and double-headed arrows represent the potential influence between components in the system. COM-B: Capability Opportunity Motivation-Behaviour; RDIS: Rubber Dam Isolation Survey.

A pre-validated questionnaire [3] in English entitled Rubber Dam Isolation Survey (E-RDIS) with some additional questions on demographic data was distributed by a single investigator in January 2022 through an online platform to dental students and interns of the dental college at Jazan University. A Google form digital version of the questionnaire was used to survey the students through emails and social media platforms (WhatsApp, Meta Platforms) to 275 prospective participants in order to meet the required sample size. Every participant signed a consent form that was sent online along with the questionnaire. The questionnaire consisted of 11 questions in total (Appendix A1); five questions were about the barriers in rubber dam application, two questions about the outcome behaviour, and four questions about the demographics. The questionnaire was based on the COM-B model of behavioural change wheel (Figure 1) [9].

**Figure 2:**
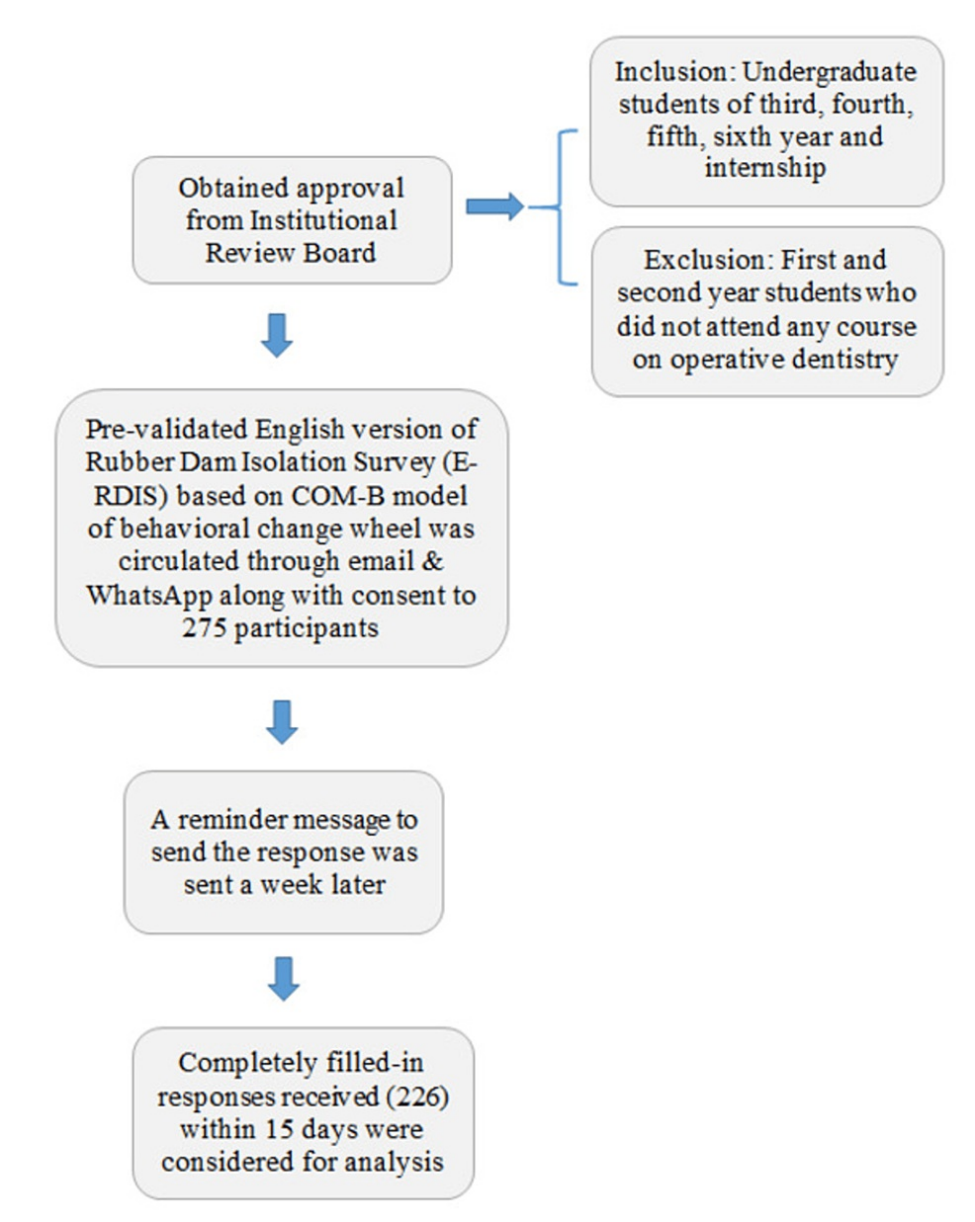
Flow chart of the data collection process from the participant COM-B: Capability Opportunity Motivation-Behaviour. This image is created by the authors.

The questions need to be responded to on a 5-point Likert scale, where 1 doesn’t fulfill the criteria, and 5 fulfills the criteria. The question about opportunity had a subdivision of two questions that assessed the importance of using rubber dam (relevance) and the availability of rubber dams in the school clinics (resources). A reminder message was sent a week later to ensure maximum response. A fixed time frame of 15 days was considered for receiving the response from the participants. The responses that were completely filled and received before the stipulated time were considered for analysis. The entire methodology has been explained in Figure 2.

## Results

Among the 275 students approached, 226 students from Jazan Dental College participated in the study resulting in an 82.2% response rate. The age range of many participants was between 21 and 30 years old (n=221) (Table 1).

**Table 1:** Demographic distribution of the participants (N = 245)

Demographic Variables		N (%)
Gender	Male	140 (57.1)
	Female	105 (42.9)
Class year	3^rd^ year	96 (39.2)
	4^th^ year	34 (13.9)
	5^th^ year	30 (12.2)
	6^th^ year	44 (18.0)
	Intern	41 (16.7)
	Saudi	197 (79.1)
	Non-Saudi	52 (20.9)
Age	21-30 years old	237 (96.7)
	41- 60 years old	1 (.4)
	Below 20 years	7 (2.9)
University	Jazan	227 (92.7)
	Other	18 (6.3)

Table 2 explains the results of the participant’s responses analyzed using the Kruskal-Wallis test. The mean students’ satisfaction with training was higher among sixth year and interns than others (Mean=3.57 and 3.56 respectively, Median=2, IQR=-1, p<0.001). Regarding capability, fourth year dental students scored higher than other groups (Mean=3.18, Median=1, IQR=1, p<0.001). Similarly, more third year students considered the use of rubber dam as an opportunity-relevance (Mean=2.93, Median=1, IQR=1, p=0.014) and opportunity-resources (Mean=3.42, Median=1, IQR=1, p<0.003) than other groups. Regarding motivation, fourth year students were more motivated to use rubber dams (Mean=4.21, Median=2, IQR=5, p=0.004) and more likely to use rubber dams in posterior teeth than others (Mean=3.74, Median=2, IQR=1, p<0.001). Also, third year students were more likely to use rubber dams in anterior teeth than other groups (Mean=3.52, Median=2, IQR=1, p<0.001).

**Table 2:** Kruskal-Wallis test of students’ response means by class years E-RDIS: English version of Rubber Dam Isolation Survey.

*E-RDIS items (N = 226)	3^rd^ year	4^th^ year	5^th^ year	6^th^ year	Dental intern	p-value
	Mean ± SD	Mean ± SD	Mean ± SD	Mean ± SD	Mean ± SD	
Satisfaction with training	1.41 ± .494	1.35 ± .485	1.37 ± .490	1.61 ± .493	1.63 ± .488	< .001>
Capability	1.38 ± .487	1.38 ± .493	1.43 ± .504	1.48 ± .505	1.39 ± .494	< .001>
Opportunity-relevance	1.43 ± .497	1.44 ± .504	1.27 ± .450	1.43 ± .501	1.29 ± .461	.005
Opportunity-resources	1.57 ± .497	1.27 ± .448	1.37 ± .490	1.43 ± .501	1.44 ± .502	.004
Motivation	2.23 ± 1.57	2.27 ± 1.88	1.93 ± 1.98	1.60 ± 1.21	1.29 ± 1.45	.003
Behavior-anterior teeth	1.58 ± .496	1.53 ± .507	1.17 ± .379	1.21 ± .408	1.46 ± .505	< .001>
Behavior-posterior teeth	1.52 ± .502	1.62 ± .493	1.63 ± .490	1.36 ± .487	1.54 ± .505	< .001>

Spearman rho correlation describes that the items of the questionnaire were significantly correlated internally ranging from strong (0.633) to weak (0.170) correlation. The internal consistency of the seven questionnaire items was very good using Cronbach’s alpha test (a=0.819) (Table 3).

**Table 3:** Spearman rho correlation between different domains of the questionnaire Spearman rho test is significant at * p < .05, ** p < .01

RDIS items (N = 249)	1	2	3	4	5	6	7
1. Satisfaction with Training	1.00						
2. Capability	.708^**^	1.00					
3. Opportunity Relevance	.364^**^	.247^**^	1.00				
4. Opportunity Resources	.216^**^	.216^**^	.252^**^	1.00			
5. Motivation	.281^**^	.241^*^	.310^**^	.169	1.00		
6. Behavior (anterior teeth)	.192^**^	.263^**^	.299^**^	.211^**^	.585^**^	1.00	
7. Behavior (posterior teeth)	.164^*^	.256^**^	.228^**^	.155^**^	.624^**^	.491^**^	1.00

In Table 4, regarding the rubber dam usage for anterior teeth, the capability to perform the procedure was a significant predictor (OR = 6.68, CI 95% = 2.33, 19.18, p <0.05) as well as, motivation to use rubber dam (OR = 12.08, CI 95% = 2.61, 55.86, p <0.05). Regarding the odds for the use of rubber dam on posterior teeth, capability (OR = 4.28, CI 95% = 1.80, 10.19, p <0.05), opportunity relevance (OR = 2.34, CI 95% = 1.09, 5.03, p <0.05) and motivation (OR = 3.74, CI 95% = 1.45, 9.64, p <0.05) were the significant predictors. Satisfaction was a barrier towards the use of the rubber dam in posterior teeth (OR = 0.200, CI 95% = 0.063, 0.627, p <0.05).

**Table 4:** Logistic regression: the use of rubber dam according to position on the jaw OR: Odds ratio, Logistic regression is significant at p < 0.05*, (none and rare =0).

Variable	Category	OR	CI 95%	
Use of rubber dam on Anterior teeth is the outcome variable				
Satisfaction		0.923	0.512	1.66
Capability		1.68	0.993	2.83
Opportunity relevance		0.901	0.553	1.47
Opportunity resources		0.759	0.455	1.27
Motivation		2.64*	1.71	4.07
Gender	Males	0.727	0.266	1.99
	Females	Reference		
Use of rubber dam on Posterior teeth is the outcome variable				
Satisfaction		0.605	0.294	1.25
Capability		1.49	0.776	2.84
Opportunity relevance		1.22	0.701	2.11
Opportunity resources		1.26	0.714	2.21
Motivation		3.61*	2.20	5.91
Gender	Males	0.520	0.168	1.61
	Females	Reference		

## Discussion

This study aimed to assess the barriers to the implementation of rubber dam isolation in restorative treatments among dental students using a validated E-RDIS questionnaire. The results of the survey helped in identifying the components, which require modification to change the student behaviour [11,12]. Previous studies posit that the barriers in using rubber dam isolation technique among dental professionals begins from their dental school during clinical practice [7,13]. Some of these barriers include patient discomfort, high cost of materials and equipment, and insufficient training and time [14,15]. Pradeep et al., 2022, reported that scarcity in appropriate clamps and denial by patients for rubber dam application are the barriers among undergraduate dental students [4]. Another study conducted among dental students by Abuzaneda BM 2021 reported that rubber dam application extends the treatment time but improves the durability of the dental fillings [16]. The most significant predictor found in this study was opportunity related to resources availability. In this questionnaire that was based on COM-B model, each component was assessed by a single item, excluding the behaviour of student. There were two separate questions for evaluating the behaviour on usage of rubber dam in anterior and posterior teeth. It also included an additional auxiliary question that focused on the satisfaction with training. It was found that the satisfaction with training differed among the students of different years. Similarly, the belief of resource availability for rubber dam usage, and the importance of rubber dam usage in affecting grades were other factors that varied among students of different class. There could be many factors that have caused the variation in predictors among the students of different years. One is self-efficacy theory, that explains that people perform task that they believe are capable to perform, whereas, they leave those in which they judge themselves to be incompetent [17]. Self-efficacy when reaches at a certain threshold does not inhibit behaviour, however, a greater frequency in behaviour is not achieved with additional gains in self-efficacy.

Studies have reported that undergraduate students receive adequate educational training on rubber dam placement and its advantages but lack hands-on training [18-20]. It has also been studied, that many variables influence undergraduate students in how they construe their careers, which includes the grades received and/or the knowledge attained [21]. Accordingly, students were more interested in achieving good grades or are more focused on learning the course material [21]. Therefore, the learning orientation among the students differs along with their plans to pursue post-graduation studies or specializations [22]. Based on the COM-B model, among the students, motivation for grades is a silent barrier for usage of rubber dam, while another important barrier as was found in this study could be opportunity-relevance.

Compared to the anterior teeth, posterior teeth were more commonly treated with rubber dams. Regarding the area of placement of rubber dam, the results reported that posterior teeth (Mean=3.74) were more frequently preferred than the anterior teeth (Mean=3.52). Similarly, previous studies found that the students were more inclined to the use of rubber dam for posterior composites and less on anterior restorations [12,13]. There were wealthy of literature outlining the reasons for not utilizing rubber dam including cost, time consuming, difficulty in placement, patient acceptance, visibility and so on [23,24]. However, usage of rubber dam placement in posterior teeth rather than anterior teeth depends on the choice of restorative material, tooth location and type of restorative procedure [7, 23]. To substantiate, Gilbert et al. 2010 has reported arguable number of studies stating that rubber dam usage was uncommon because of above mentioned factors, with few dentists prefer using rubber dam for class 2 amalgam restorations and composite restoration on posterior teeth [23]. The other possible reason could be the property of rubber dam itself, as it is efficient in decreasing salivary contamination and maintains a dry working field which is otherwise arduous in the posterior region when compared with the anterior teeth [25,26].

Limitations of the study

Firstly, only one item assessed each component of COM-B model; however, it made the survey completion easy. Secondly, there could be some recall bias, as the data collected were self-reported by the students. Thirdly, the responses of third year students who only have preclinical experience could not be used to extrapolate the behaviour of other students with clinical experience about the use of rubber dam.

Strengths of the study

However, the results of the study can be used by the stakeholders, in understanding what factors among students are causing barriers in implementation of rubber dam isolation for adhesive restorative treatment. This in turn can help them in implementing targeted strategies for removing these barriers, and enhancing positive behaviour towards its usage. One such approach could be to emphasize teaching rubber dam technique to students and encouraging their learning by making it a required subject for grades. This could increase the use of rubber dam among undergraduate students for all restorative procedures.

A systematic review from the Cochrane database reported that there is a need for high quality research and clinical evaluation to validate the influence of rubber dam isolation on the survival of restoration [25]. Further studies should focus on evaluating the quality and longevity of the restoration that is performed by undergraduate students with and without the application of rubber dam during adhesive restorative treatments. This could also improve the patient acceptance towards rubber dam application for better longevity of restorations.

## Conclusions

The E-RDIS is a validated tool to understand the barriers in rubber dam isolation behavior for adhesive restorative treatments among dental students. The most significant barrier found was the motivation in the form of grades to encourage the use rubber dam. The satisfaction with training differed among the students of different years. Similarly, the confidence level of students and their belief of resource availability for rubber dam usage were other factors that varied among students of different years. Increased application of rubber dam technique could result from emphasizing its importance in grades and emphasizing students' ability to perform the technique.

## Appendices

**Table 5:** Rubber Dam Isolation Survey (RDIS) for adhesive restorative treatments Adhesive Restorative Treatment refers to resins, glass ionomers, and sealants. Instructions to the participants: Please answer all of the following questions by selecting from the multiple choices. Choose one answer only.

S. No	Questions	Choices
1	How satisfied are you with the training (theoretical/practical) you have received whilst studying dentistry regarding the use of rubber dam isolation?	Not at all satisfied
		Slightly satisfied
		Moderately satisfied
		Very satisfied
		Completely satisfied
2	How important is rubber dam isolation in terms of effectiveness/quality of your adhesive restorative treatment?	Not at all important
		Slightly important
		Moderately important
		Very important
		Extremely important
3	Do you have all the resources you need (time, instruments, materials, etc.) in order to achieve rubber dam isolation?	Never
		Rarely
		Sometimes
		Most of the time
		Always
4	How does your use of rubber dam isolation for adhesive restorative treatments affect your grade?	Not at all
		Slightly
		Moderately
		Very much
		Completely
		I don't know
5	How often do you use rubber dam isolation for adhesive restorative treatments of the anterior teeth?	Never
		Rarely
		Sometimes
		Most of the time
		Always
6	How often do you use rubber dam isolation for adhesive restorative treatments of the posterior teeth?	Never
		Rarely
		Sometimes
		Most of the time
		Always
7	How old are you today?	Below 20 years
		21 to 30
		Above 30 years
8	Gender	Male
		Female
9	University	Jazan University
		Others. Please: specify: _______________________
10	Education	Dental intern
		3^rd^ year
		4^th^ year
		5^th^ year
		6^th^ year
